# Displacement of Abdominal Organs Into the Thoracic Cavity: A Rare Case of Adult Bochdalek Hernia

**DOI:** 10.7759/cureus.56950

**Published:** 2024-03-26

**Authors:** Sien Hu, Xiaohui Yang, Yulian Wu

**Affiliations:** 1 Department of Surgery, Fourth Affiliated Hospital, Zhejiang University School of Medicine, Yiwu, CHN; 2 Department of Surgery, Second Affiliated Hospital, Zhejiang University School of Medicine, Hangzhou, CHN

**Keywords:** ct, patch repair, intrathoracic kidney, bochdalek hernia, congenital diaphragmatic hernia

## Abstract

Congenital diaphragmatic hernias are primarily found in infants and have a high mortality rate due to neonatal respiratory distress. The most common type of congenital diaphragmatic hernia is Bochdalek hernia, which occurs in the posterolateral diaphragm, with the left side being the most commonly affected. However, congenital diaphragmatic hernias are extremely rare in adults and are often misdiagnosed due to their subtle symptoms. Therefore, we suggest that a contrast-enhanced CT scan should be used for early screening and diagnosis in all patients with sudden severe pain or recurrent ambiguous symptoms in the chest and abdomen. This case report presents a rare occurrence of Bochdalek hernia in an adult male. The patient experienced nonspecific abdominal symptoms after eating. The hernia resulted in the displacement of the left kidney, the transverse colon of the splenic flexure, and most of the stomach into the thoracic cavity. This displacement led to atelectasis of the left lung, which reached three-fifths of its capacity. The patient underwent successful treatment using a combination of laparoscopy and open surgery. Follow-up CT scans conducted two weeks, three months, and one year later revealed a stable condition with no complications.

## Introduction

Congenital diaphragmatic hernia (CDH) occurs in approximately 1 in every 3,000 live births and is extremely rare in adults [1]. CDH can be classified into three types based on their anatomical location, namely, posterolateral, anterior (or Morgagni), and central. The latter two are collectively referred to as non-posterolateral hernias. Anterior hernias account for 23%-28% of cases, while central hernias account for 2%-7%. Posterolateral hernias are the most common type, comprising 70%-75% of cases. Among these, 85% occur on the left side, 13% on the right side, and 2% are bilateral [2,3]. Bochdalek hernias, which involve the passage of abdominal organs into the thoracic cavity through a diaphragmatic defect, have a high mortality rate. It is extremely rare for abdominal organs such as kidneys to be located within the thoracic cavity. In fact, the occurrence of an intrathoracic kidney is quite rare, appearing in only 1 in 10,000 cases. It is most commonly found on the left side (61%) and is more prevalent in males (1.7:1) [4,5]. This rare condition occurs due to a closure defect at the end of the embryonic stage when the posterolateral portion of the pleuroperitoneal canal fails to close during development. While most cases are symptomatic in the neonatal period [6,7], it is often insidious in adults and is incidentally diagnosed in 5% of visits for abdominal or respiratory problems [6,8]. Bochdalek hernia is more common in adult male patients (62%) [9,10], and, in some cases, the hernia may be asymptomatic and detected incidentally during an imaging examination. Therefore, the exact prevalence of Bochdalek hernia in adults cannot be determined.

This case report presents a rare clinical case of Bochdalek hernia with an intrathoracic ectopic kidney in an adult patient. Additionally, the options for diagnosing and treating this rare disease in adults are also discussed.

## Case presentation

A 32-year-old man presented to the emergency department complaining of worsening pain in his left upper abdomen and left renal area for the past 12 hours. He had been experiencing discomfort in the same area for the past three years. The patient reported sudden and persistent cramps under the left diaphragm after eating, without any other accompanying symptoms. As part of the routine examination, an emergency physician performed a CT scan to investigate the abdominal symptoms. The scan revealed the presence of a left diaphragmatic hernia, with the left kidney, part of the stomach, and bowel located within the chest cavity.

During the treatment, the doctor provided symptomatic treatment; however, it did not yield favorable outcomes. After five hours, the pain spontaneously resolved and the patient was able to return home without any symptoms thereafter.

**Figure 1:**
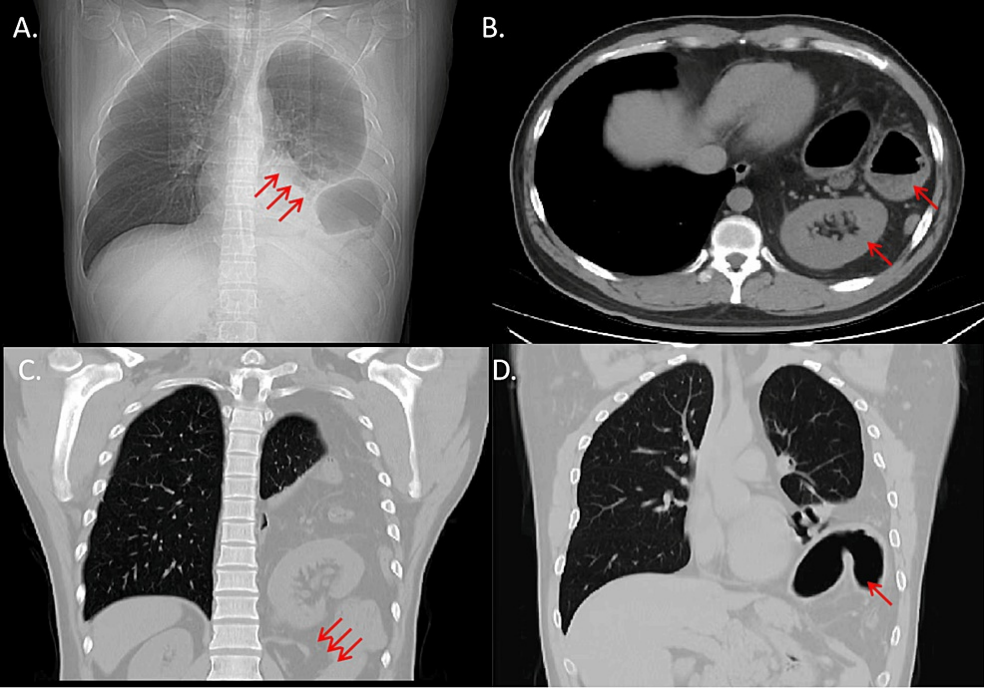
The Imaging examination showing a left diaphragmatic hernia with the presence of a left kidney, part of the stomach, and bowel within the chest cavity. Contraction and partial consolidation of the left lower lobe, along with elevation of the left diaphragm (Figure 1A, arrow). A defect in the posterolateral aspect of the left hemidiaphragm (Figure 1C, arrow). Herniation of the left kidney, part of the stomach, and intestines into the chest cavity (Figure 1B, arrow; Figure 1D, arrow).

One month later, the patient considered surgery as a means to completely cure the diaphragmatic hernia. Upon physical examination after hospitalization, it was observed that the inferior boundary of the left lung had risen to the fifth intercostal space at the midaxillary line. The chest CT images indicated contraction and partial consolidation of the left lower lobe, along with elevation of the left diaphragm (Figure 1A, arrow). A defect in the posterolateral aspect of the left hemidiaphragm was also observed (Figure 1C, arrow), leading to the herniation of the left kidney, part of the stomach, and intestines into the chest cavity (Figure 1B, arrow; Figure 1D, arrow). Pulmonary function tests diagnosed the patient with moderate mixed ventilatory dysfunction, although they did not exhibit any respiratory symptoms. Other laboratory tests, such as urinalysis, routine blood tests, sexually transmitted disease tests, and electrocardiogram, yielded normal results.

After conducting a preoperative consultation with the patient, we determined that either laparoscopic or open surgery would be necessary to complete the procedure. Our initial approach was to use the minimally invasive laparoscopic method. During the laparoscopic exploration, we discovered a fan-shaped defect measuring approximately 8 × 6 cm on the left posterior side of the diaphragm. This confirmed the protrusion of various organs, including the stomach, duodenum, kidney, part of the transverse colon, colon splenic flexure, and herniation of the greater omentum. However, due to the high position of the defect and its large size, laparoscopy alone was insufficient to fully retract and secure the herniated abdominal organs for further procedures. As planned, we decided to convert the surgery to an open procedure. For the open surgery, we made a left subcostal incision. After carefully separating the adhesions around the hernia ring, the defect became visible once the hernia contents were placed back. We used an anti-adhesion patch to cover the defect and sutured the mesh to the diaphragm and pleura, securing it with glue. Finally, we successfully restored the spleen, stomach, kidney, colon, and small intestine to their normal anatomical structures. The entire process took approximately 3.5 hours.

**Figure 2:**
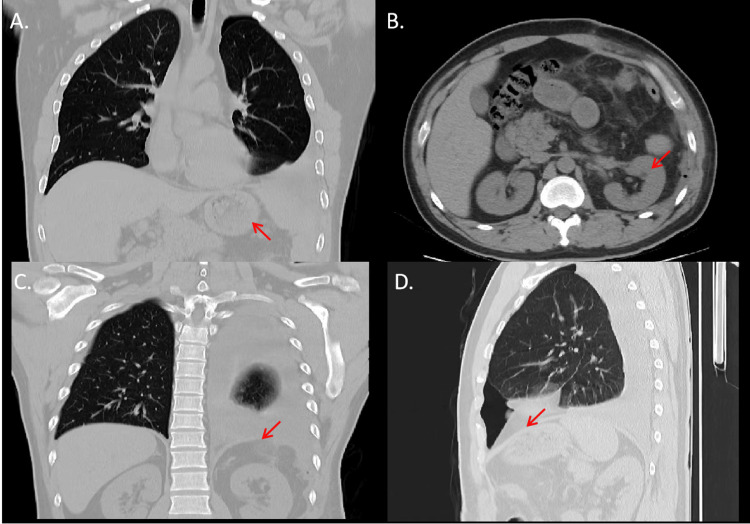
Chest CT at the two-week follow-up showing the left lower lung, the abdominal organs, and satisfactory repair. The abdominal organs returning to the abdominal cavity (Figure 2A, arrow; Figure 2B, arrow). The effect of patch repair of the diaphragm (Figure 2C, arrow, Figure 2D, arrow).

**Figure 3:**
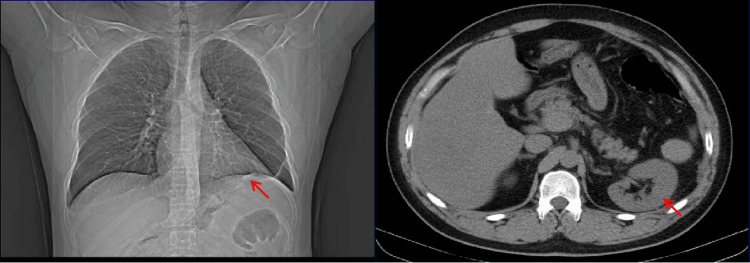
Chest CT at the three-month follow-up. Notable expansion of the left lower lung and the secure fixation of abdominal organs within the abdominal cavity (arrow).

**Figure 4:**
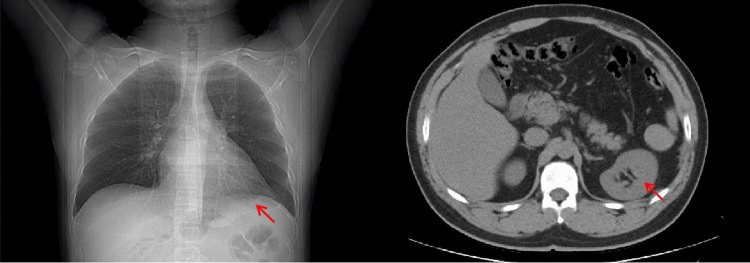
Chest CT at the one-year follow-up. Notable expansion of the left lower lung and the secure fixation of abdominal organs within the abdominal cavity (arrow).

The patient exhibited a successful recovery after undergoing surgery. The patient had his first follow-up visit two weeks after the operation. Chest CT showed that the abdominal organs in the chest cavity had returned to the abdominal cavity and the repaired diaphragm (Figure 2, arrow). Subsequent chest CT scans conducted three months (Figure 3) and one year (Figure 4) post-surgery revealed a notable expansion of the left lower lung, accompanied by the secure fixation of abdominal organs within the abdominal cavity. Furthermore, the patient experienced relief from abdominal discomfort following the surgical procedure.

## Discussion

Bochdalek hernia was first described by Vincenz Alexander Bochdalek (1801-1883), a Czech anatomist and pathologist, in 1848, and it is named after him [11]. In children, Bochdalek hernia often presents with acute respiratory failure, while it is rare in adults. In adults, it typically manifests as atypical chronic abdominal and respiratory symptoms, such as abdominal pain, loss of appetite, chest pain, cough, and dyspnea [12,13]. Our patient experienced only chronic abdominal discomfort but had a sudden onset of left subphrenic cramps after eating. It is speculated that this may be due to the increased volume of the gastroduodenum after eating, which causes hernia stenosis. Although CT scans revealed atelectasis on the left side, the patient did not exhibit any respiratory symptoms.

Regarding the physical examination, there are limited case reports in the literature that discuss relevant cases of physical examination. Of these reports, only one mentioned that the abdominal signs were completely normal [12]. It is noteworthy that our patient also exhibited completely normal abdominal signs. However, changes in chest signs, such as the upward movement of the lower lung border and the weakening of breath sounds, were observed during the physical examination of the chest. These signs are not considered characteristic and may contribute to the high rate of misdiagnosis.

The radiology reports are the primary diagnostic method for hernias. While numerous studies have reported left-sided cases, only one study found that 68% of adult cases had right-sided hernias compared to 18% and 14% for left-sided and bilateral hernias, respectively. This suggests that symptomatic cases are more likely to occur on the left side, while asymptomatic patients are usually on the right side, possibly due to the liver’s barrier function against hernias [14]. In neonates, approximately 78% of defects were left-sided, 21% were right-sided, and 1% were bilateral [15].

Chest radiographs can be used for diagnosis, but it is important to note that the findings can sometimes be confused with other conditions such as pulmonary sequestration, atelectasis, or consolidation. Without a CT scan, there is a risk of misdiagnosing up to 38% of Bochdalek hernias as pleural effusion, empyema, or pneumothorax [16]. Therefore, the preferred examination modality is contrast-enhanced CT of the chest [6-10,12-14,16-18]. This type of examination provides a better understanding of the thoracic contents and allows for visualization of diaphragmatic defects. Excretory urography can also be used to confirm the presence of an intrathoracic kidney. However, it is important to note that a normal CT image does not completely rule out the diagnosis of diaphragmatic hernia. In cases of left diaphragmatic defect, this method has shown a sensitivity of 78% and a specificity of 100% [13,17].

Conservative treatment of Bochdalek hernia is currently not recommended. If left untreated, complications such as gastric perforation, gastrointestinal obstruction, volvulus, spleen rupture, and pneumothorax may occur. Therefore, surgery to repair the diaphragm defect is recommended [12,16,18]. Bochdalek hernias can be surgically repaired using thoracotomy, laparotomy, thoracoscopy, or laparoscopy [12,13,16,18]. There is literature suggesting that repairing the diaphragm through a transthoracic approach (i.e., lateral thoracotomy) is easier than a transabdominal approach. However, in patients with signs of obstruction or strangulation, laparoscopic or exploratory laparotomy is preferred [19]. Choosing a transabdominal approach also has the advantage of a lower morbidity rate compared to open thoracotomy [18]. There is literature suggesting that laparoscopy is generally considered more beneficial than standard open surgery due to its less invasive nature, reduction in postoperative pain, and ability to make more precise incisions [20]. However, in cases where there are multiple ectopic organs herniated into the thoracic cavity, it may be necessary to convert to open abdominal surgery to pull them back into the abdominal cavity for fixation [13,16]. According to reports in the literature, for Bochdalek hernias with a diameter of 4 cm and no organ herniation, a one-stage suturing technique under laparoscopy is used [20]. When the diameter of the diaphragm defect reaches 5 cm and one or more organs are herniated, laparoscopic treatment is initially preferred, but eventually, laparotomy and mesh repair must be performed [13,16]. In cases where the diaphragm defect area is small, intermittent non-absorbable sutures can be used for primary sutures. However, when the diaphragm defect area exceeds 20-30 cm^2^, mesh should be employed [12,13,16]. Initially, it was planned to repair the defect using laparoscopic technique. However, due to the large size of the defect, its high position, and the inability to pull back the hernia contents into the abdominal cavity during laparoscopy, it was decided to switch to laparotomy after intraoperative discussion. Therefore, while there are various surgical methods to choose from, it is important to have a preparatory plan and design an individualized surgical plan for each case.

In the case of an intrathoracic kidney, we opted to relocate it to the abdominal cavity due to its abnormal position and the potential for complications. Based on the available literature, surgical intervention is not necessary if there are no other renal pathological features; however, close monitoring and follow-up are required for patients. Some authors also suggest considering surgical repair [21].

## Conclusions

The case presented in this report examines the options for examination and surgical treatment of Bochdalek hernia. It is important to note that this report is based on a single clinical case and cannot be generalized to all cases of Bochdalek hernia. However, even for experienced clinicians, diagnosing CDH in adults is rare and challenging due to atypical symptoms. Therefore, it is crucial to promptly diagnose Bochdalek hernia and maintain a high level of clinical suspicion to prevent complications. CT scan is the recommended screening and diagnostic examination. Surgical repair is advised for all cases, and the surgical plan should be tailored to each individual patient.
